# Genome-wide pleiotropy and shared biological pathways for resistance to bovine pathogens

**DOI:** 10.1371/journal.pone.0194374

**Published:** 2018-04-02

**Authors:** M. Mahmoud, Y. Zeng, M. Shirali, T. Yin, K. Brügemann, S. König, C. Haley

**Affiliations:** 1 Institute of Animal Breeding and Genetics, Justus-Liebig-University Gießen, Gießen, Germany; 2 MRC Human Genetics Unit, MRC Institute of Genetics and Molecular Medicine, University of Edinburgh, Edinburgh, United Kingdom; 3 Centre for Clinical Brain Sciences, University of Edinburgh, Kennedy Tower, Royal Edinburgh Hospital, Edinburgh, United Kingdom; 4 Roslin Institute and Royal (Dick) School of Veterinary Studies, University of Edinburgh, Midlothian, United Kingdom; University of Bern, SWITZERLAND

## Abstract

Host genetic architecture is a major factor in resistance to pathogens and parasites. The collection and analysis of sufficient data on both disease resistance and host genetics has, however, been a major obstacle to dissection the genetics of resistance to single or multiple pathogens. A severe challenge in the estimation of heritabilities and genetic correlations from pedigree-based studies has been the confounding effects of the common environment shared among relatives which are difficult to model in pedigree analyses, especially for health traits with low incidence rates. To circumvent this problem we used genome-wide single-nucleotide polymorphism data and implemented the Genomic-Restricted Maximum Likelihood (G-REML) method to estimate the heritabilities and genetic correlations for resistance to 23 different infectious pathogens in calves and cows in populations undergoing natural pathogen challenge. Furthermore, we conducted gene-based analysis and generalized gene-set analysis to understand the biological background of resistance to infectious diseases. The results showed relatively higher heritabilities of resistance in calves than in cows and significant pleiotropy (both positive and negative) among some calf and cow resistance traits. We also found significant pleiotropy between resistance and performance in both calves and cows. Finally, we confirmed the role of the B-lymphocyte pathway as one of the most important biological pathways associated with resistance to all pathogens. These results both illustrate the potential power of these approaches to illuminate the genetics of pathogen resistance in cattle and provide foundational information for future genomic selection aimed at improving the overall production fitness of cattle.

## Introduction

Infection is the colonization of the host by at least one domain of pathogens such as viruses, bacteria, fungi, and/or parasites (parasite infestation). Each type of pathogen is different in its invasion and replication in the host tissue, namely “infectivity” and/or the capacity to pass from one individual to another, namely “transmissibility” [1, 2]. Although variation in resistance to individual infectious agents (pathogens) is associated with levels of immunisation, disease treatment policies, diet and other environmental factors, previous studies of the resistance to various pathogens in animals [3] and humans [4] have also revealed a major role of host genetic factors in pathogen resistance and host survival.

Host genetic architecture in cattle has been found to be a major factor in resistance to multifactorial diseases and disorders such as infertility, metabolic disorder, claw disorder and mastitis [5]. Several studies have addressed the importance of the genetic contribution of host resistance/susceptibility to different domains, species, and subspecies of pathogens [6, 7]. The most studied pathogen subspecies in the field of dairy cattle breeding (particularly in Europe) are *Mycobacterium avium* subspecies *Tuberculosis* [8–14], and *Mycobacterium avium* subspecies *Paratuberculosis* [15–18]. Another important pathogen affecting calves is *Salmonella typhimurium*. Wray and Sojka [19] reported some phenotypic variation in resistance to Salmonella between Jersey calves and Friesian calves, which may be due to the genetic variation among breeds. Templeton et al., [20] noted that calves of sires with a high resistance to Brucellosis also show a high resistance to Salmonella, suggesting a genetic contribution to the resistance to this pathogen. In studies of the genetics of resistance to viral pathogens, resistance has mostly been measured indirectly as the symptoms of infection in animals, rather than directly measured as susceptibility to the pathogen itself. For instance, in 2008, Heringstad et al., [21] estimated the heritability of susceptibility to respiratory diseases to be 0.05±0.018. Estimation of genetic variation underlying the resistance/susceptibility for parasitic infestation has been well studied in small ruminants [22], and to a small extent in cattle [23]. In Canadian Holstein, the heritability for susceptibility to *Neospora caninum* was in a range between 0.08±0.02 and 0.12±0.04 [24]. In Dutch Holstein-Friesian, the heritability of eggs/larvae count in animal feces was estimated to be from 0.00±0.02 to 0.25±0.05 [25]. The resistance to ectoparasites in cattle was studied and reviewed by [26] who concluded that the heritability of resistance to ticks was 0.31, and 0.21 for resistance to Buffalo flies.

A number of studies have also detected quantitative trait loci (QTLs) associated with susceptibility/resistance to infection diseases in farm animals. Lee et al., [27] found 11 QTLs on three chromosomes (BTA15, BTA17, and BTA22), significantly associated with susceptibility to Foot-and-mouth disease in Holstein cattle. Casas et al., [28] searched for markers directly associated with the susceptibility to infection with Bovine viral diarrhea virus in feedlot cattle, and found a significant association on Chromosome BTA14.

Despite estimates of the heritability of resistance for some pathogens, genetic correlations (pleiotropy) among resistance between these domains of pathogens are still unknown, as is the level of pleiotropy among resistance to different species and subspecies within each domain of these pathogens. Several reasons lie behind the absence of the genetic correlations among resistance/susceptibility to various pathogens. First, the most popular method used in animal breeding for estimating genetic correlation has been the pedigree-base restricted maximum likelihood approach (Pedigree-REML). This method tests genetic overlap of traits between related individuals within pedigrees. To quantify the genetic correlation between traits in a family-based study, we may need to measure the traits in individuals with pedigree relationships [29]. Consequently, it will be challenging and costly to repeat measurements on all animals, in particular for these low-prevalence traits and/or for traits where slaughter of the animals to measure their resistance is needed (i.e. endo-parasite infestation). Moreover, some disease traits (i.e. Bovine respiratory diseases) may result in death of the animal (at a young age) before it is possible to measure other traits (such as milk production after first calving) for which we want to test the correlation.

The genomics era has provided a solution to the problem of estimating genetic correlations, by allowing the genetic correlation to be estimated using genomic variants (i.e. SNPs) instead of using pedigree information, providing more precise and accurate estimates for the narrow-sense heritability ($h_{SNP}^{2}$) in case-control studies and for quantitative traits as well as for the coheritability between such traits, which in particular does not need the measurement of multiple traits per animal. So far, several different methods have been developed for estimating the (co)heritability using genomic data: the first method uses the significantly associated SNPs found in large GWAS studies to estimate the causal relationships between risk factors and disease. This method is efficient only in case of the traits with many significant SNPs, which is usually not the case in resistance/susceptibility traits. For complex traits (especially for those recorded in case control studies), it is recommended to use genome-wide data (array or sequence data) instead of using only significant markers to estimate genetic correlation [30], and this approach has been implemented in two published methods; Genomic-restricted maximum likelihood (G-REML) [31, 32] and polygenic scores [33, 34]. One of the limitations for the application of the last two methods in complex traits is the availability of individual-level genotype data. Hence, Bulik-Sullivan and colleagues [29, 35] developed LD Score regression using the GWAS summary statistics instead of the individual-level genotype data for estimating heritabilities and genetic correlations.

In this study, we use individual-level genotypes for ~ 20000 animals from cow calibration groups (NB Cow calibration groups: is a programme initiated in Germany combining information for novel traits with high-density genetic markers based on ~20,000 genotyped cows, to offer a new perspective on breeding for improved disease resistance [36]). We applied the G-REML method to estimate the heritability of the 23 resistance traits and the genetic correlations between these traits, and the correlations between the resistance traits with calf performance and cow productivity. We also implemented a post-GWAS functional analysis to estimate the pleiotropy based on different scales; scale 1) pleiotropy among all 23 resistance traits based on gene analysis; scale 2) pleiotropy among all 23 traits based on gene-set analysis (biological pathways), to understand the biological background of the underlying resistance/susceptibility to infectious diseases.

## Results

### SNP heritabilities

Using a univariate G-REML model, we estimated the SNP-based heritabilities (**h**^**2**^_**SNP**_) for all resistance, performance, and productivity traits (Table 1). SNP heritabilities of resistance to bacterial pathogens ranged from 0.03±0.01 to 0.21±0.01 in calves, and from 0.02±0.01 to 0.13±0.02 in cows. Higher SNP heritabilities were estimated for the resistance to viral pathogens, ranging from 0.16±0.03 to 0.22±0.03 in calves, and 0.10±0.01 in cows. For resistance to trichophyton (the only fungal pathogen) in calves, the heritability estimate was 0.17±0.03, and in cows it was 0.04±0.01. In parasitic infestation, the SNP heritability estimates in calves ranged from 0.04±0.01 to 0.14±0.03, while in cow’s estimates varied from 0.19±0.02 to 0.25±0.02. For calf performance traits, the SNP heritability explained 0.30±0.01 of the phenotypic variance in birthweight, while 0.08±0.02 of the phenotypic variance in average daily gain was explained. For cow productivity traits, SNPs explained 0.19±0.02 of the variance in average milk yield, and 0.25±0.02 of the variance for the fat to protein ratio trait.

**Table 1:** Number of genotyped samples and estimated SNP-based heritabilities (h^2^_SNP_) for resistance and performance traits in calves; and resistance and productivity traits in cows.

**Calf resistance traits**	**Infected**	**Resistant**	**Incidence**	**Total**	**h**^**2**^_**SNP**_**±s.e.**
Bacterial pathogens					
*Salmonella*	271	570	0.32	841	0.21±0.01
*Escherichia coli*	19	483	0.04	502	0.03±0.01
Viral pathogens					
*Bovine respiratory syn*.	143	375	0.28	518	0.16±0.03
*Bovine herpes virus 1*	113	162	0.41	275	0.22±0.03
Fungal pathogen					
*Trichophyton*	421	875	0.32	1296	0.17±0.03
Parasitic pathogens					
*Cryptosporidium*	238	749	0.24	987	0.14±0.03
*Coccidia*	270	770	0.26	1040	0.11±0.03
*Myiasis*	362	107	0.77	469	0.13±0.02
*Bovicola bovis*	20	445	0.04	465	0.04±0.01
**Calf performance traits**	**Mean±s.d.**				
Birthweight (in kg)	41.09±4.83			17976	0.30±0.01
Average daily gain (in kg)	0.77±0.17			7673	0.08±0.02
**Cow resistance traits**	**Infected**	**Resistant**	**Incidence**		
Bacterial pathogens					
*Salmonella*	103	794	0.11	897	0.11±0.02
*Escherichia coli*	87	905	0.09	992	0.08±0.02
*Staph*. *Aureus*	102	805	0.11	907	0.12±0.01
*Staph*. *Haemolyticus*	379	802	0.32	1181	0.08±0.02
*Strep*. *Agalactiae*	51	1177	0.04	1228	0.11±0.06
*Strep*. *Dysgalactiae*	18	797	0.02	815	0.02±0.01
*Strep*. *Uberis*	101	798	0.11	899	0.13±0.02
*Clost*. *Perfringens*	21	238	0.08	259	0.12±0.06
*Mycobac*. *Paratuberculosis*	30	781	0.04	811	0.10±0.05
Viral pathogen					
*Rotavirus*	237	1253	0.16	1490	0.10±0.01
Fungal pathogen					
*Trichophyton*	11	1564	0.01	1575	0.04±0.01
Parasitic pathogens					
*Dictyocaulus viviparus*	671	652	0.51	1323	0.06±0.01
*Bovicola bovis*	25	929	0.03	954	0.06±0.02
*Chorioptic scabies*	50	940	0.05	990	0.10±0.02
**Cow productivity traits**	**Mean±s.d.**				
Average milk yield (in kg)	30.83±5.51			9959	0.19±0.02
Fat to protein ratio (in %)	1.24±0.17			9959	0.25±0.02

### Pleiotropy among calf resistance traits

**Fig 1:**
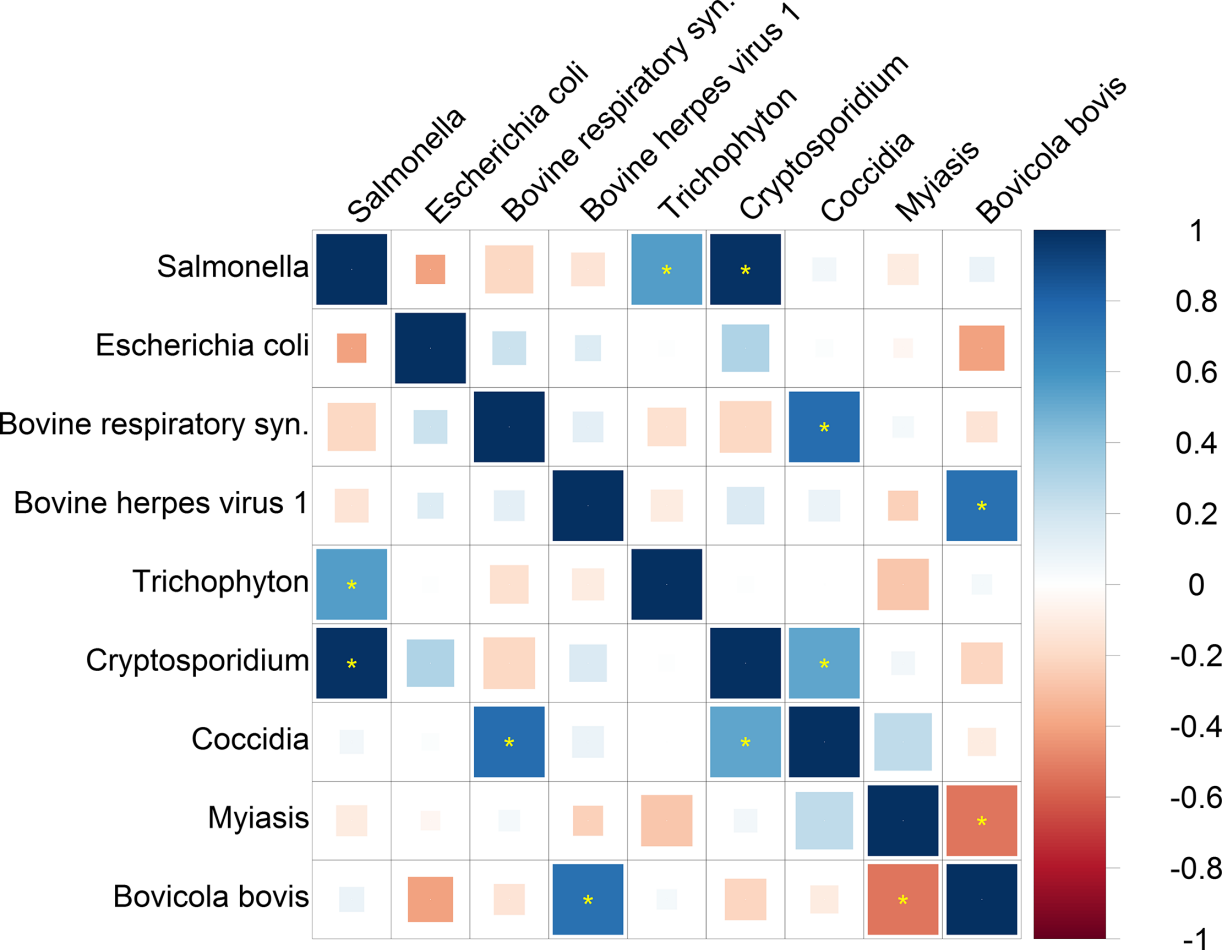
Genetic correlations among the 9 calf resistance traits analyzed by G-REML. Blue, positive genetic correlation; red, negative genetic correlation. Larger squares correspond to more significant P values. Genetic correlations that are different from zero at a false discovery rate (FDR) of 1% are shown as full-sized squares. Genetic correlations that are significantly different from zero after Bonferroni correction for the 36 tests in this analysis are marked with a yellow asterisk. We show results that do not pass multiple-testing correction as smaller squares. All genetic correlations in this report can be found in tabular form in S1 Table.

A bivariate analysis was used to estimate the level of pleiotropy among calf resistance traits using genome-wide SNPs for all 36 pairwise combinations of the nine resistance traits, shown in Fig 1 and S1 Table. The genetic correlation was significantly different from zero (according to FDR <1% and according to the Bonferroni threshold) between the resistance to Salmonella pathogen and Trichophyton pathogen (0.55±0.07), between Salmonella and Cryptosporidium (0.98±0.01), between Bovine respiratory syn. and Coccidia (0.46±0.06), between Bovine herpesvirus 1 and Bovicola bovis (0.74±0.18), between Cryptosporidium and Coccidia (0.52±0.06), and between Myiasis and Bovicola bovis (-0.54±0.06).

### The pleiotropy among cow resistance traits

**Fig 2:**
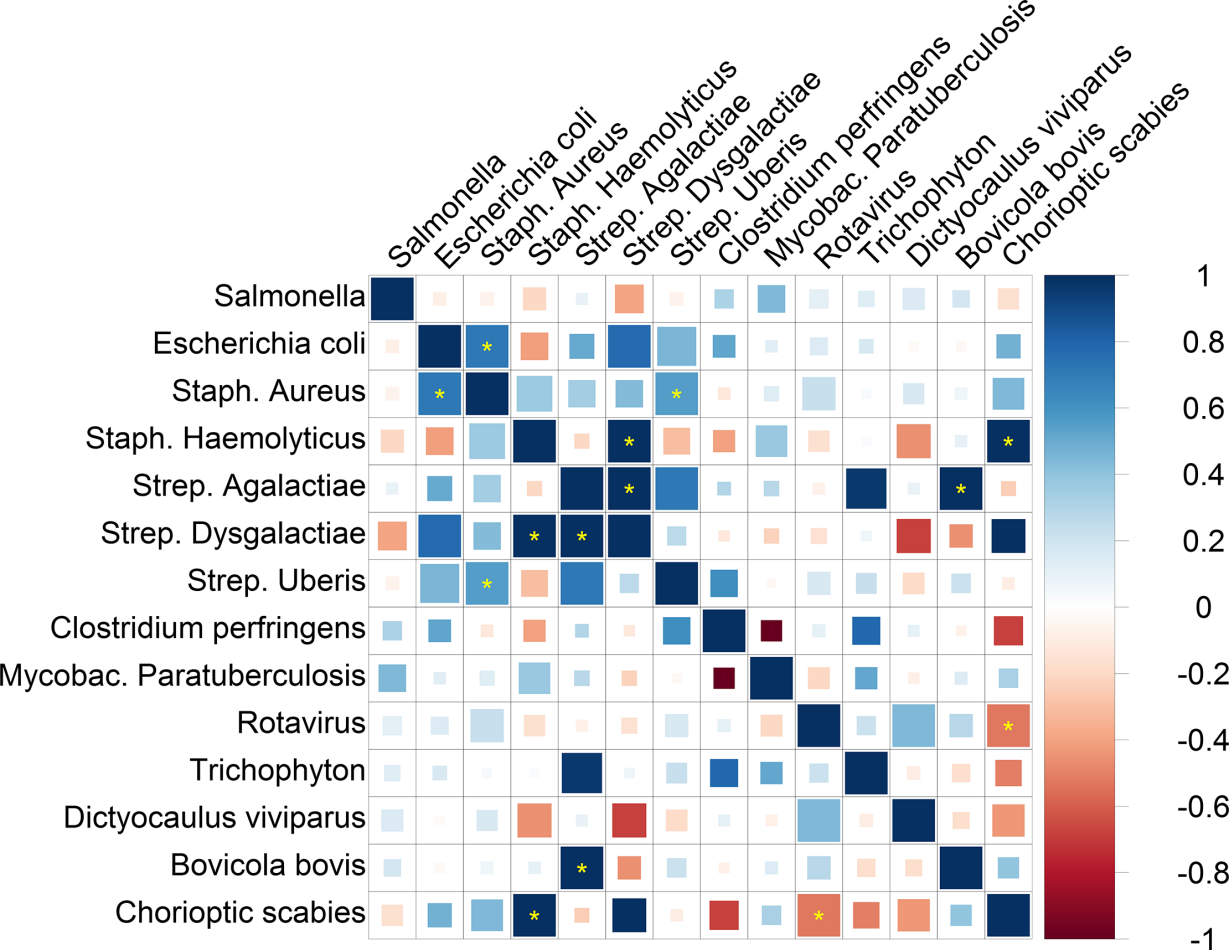
Genetic correlations among the 14 cow resistance traits analyzed by G-REML. Blue, positive genetic correlation; red, negative genetic correlation. Larger squares correspond to more significant P values. Genetic correlations that are different from zero at a false discovery rate (FDR) of 1% are shown as full-sized squares. Genetic correlations that are significantly different from zero after Bonferroni correction for the 105 tests in this analysis are marked with a yellow asterisk. We show results that do not pass multiple-testing correction as smaller squares. All genetic correlations in this report can be found in tabular form in S2 Table.

A second bivariate analysis was used to estimate the pleiotropy among cow resistance traits using genome-wide SNPs for all 105 pairwise combinations of the 14 resistance traits (all results are shown in Fig 2 and S2 Table). The estimated genetic correlation was significantly different from zero (according to FDR <1% and according to the Bonferroni threshold) between the resistance to Escherichia coli pathogen and Staph. Aureus pathogen (0.71±0.15), between Staph. Aureus and Strep. Uberis (0.56±0.10), between Staph. Haemolyticus and Strep. Dysgalactiae (1.00±0.34), between Staph. Haemolyticus and Chorioptic scabies (1.00±0.11), between Strep. Agalactiae and Strep. Dysgalactiae (1.00±0.28), between Strep. Agalactiae and Bovicola bovis (1.00±0.36), and between Rotavirus and Chorioptic scabies (-0.52±0.16). The estimated genetic correlation was significantly different from zero (with FDR <2%) between the resistance to Escherichia coli and Strep. Dysgalactiae (0.78±0.22), Strep. Agalactiae and Strep. Uberis (0.72±0.27), Strep. Agalactia and Trichophyton (0.96±0.33), and Rotavirus and Dictyocaulus viviparus (0.44±0.13).

The genetic correlation between calf and cow resistance traits was significantly different from zero for two different traits (S3 Table). The estimated genetic correlation between resistance to the Salmonella pathogen in calves and cows was -0.26±0.09 (p-value ≤ 0.001), and the genetic correlation between resistance to the Trichophyton pathogen in calves and cows was 0.18±0.12 (p-value ≤ 0.05).

### Pathogen resistance and calf performance

**Fig 3:**
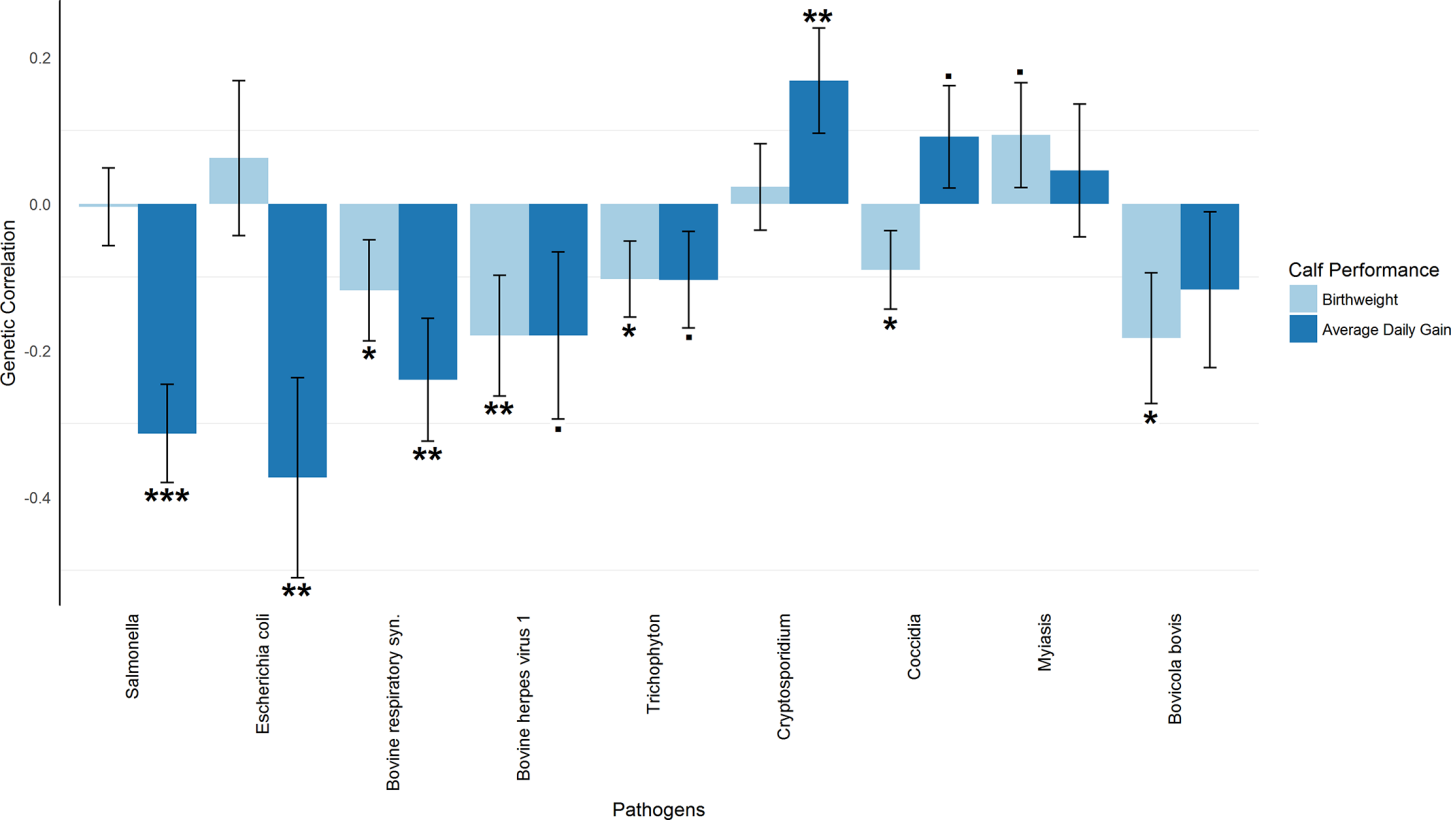
Estimated genetic correlations of birthweight (BW) and average daily gain (ADG) with all resistance traits in calves. This plot compares the genetic correlation between BW and all calf resistance traits with the genetic correlation between ADG and all calf resistance traits obtained from G-REML. The horizontal axis indicates pairs of phenotypes (BW and ADG), and the vertical axis indicates genetic correlation. Error bars represent standard errors. ‘***’P ≤ 0.001; ‘**’P ≤ 0.01; ‘*’P ≤ 0.05; ‘·’P ≤ 0.1.

The genetic correlations of birthweight with the resistance to viral, fungal, and parasitic (except for Cryptosporidium) pathogens were highly significantly negative for resistance to Bovine herpes virus 1 (P ≤ 0.01), and also significantly negative (P ≤ 0.05) for resistance to the Bovine respiratory syn., Trichophyton, Coccidia and Bovicola bovis pathogens. There were no significant positive correlations between birthweight and resistance, although that with Myiasis approached significance (P ≤ 0.1). The genetic correlation of average growth rate was very highly significantly (P ≤ 0.001) negative with resistance to Salmonella, highly significantly negative with resistance to the Escherichia coli and Bovine respiratory syn. Pathogens, and negative and approaching significance (P ≤ 0.1) with resistance to Bovine herpes virus 1 and Trichophyton pathogens. Positive correlations with average daily gain were only significant for Cryptosporidium (P ≤ 0.01) and approaching significance for Coccidia (P ≤ 0.1) (Fig 3). All correlations in calf performance traits had standard errors that ranged from 0.05 to 0.14 (S4 Table).

### Pathogen resistance and cow productivity

**Fig 4:**
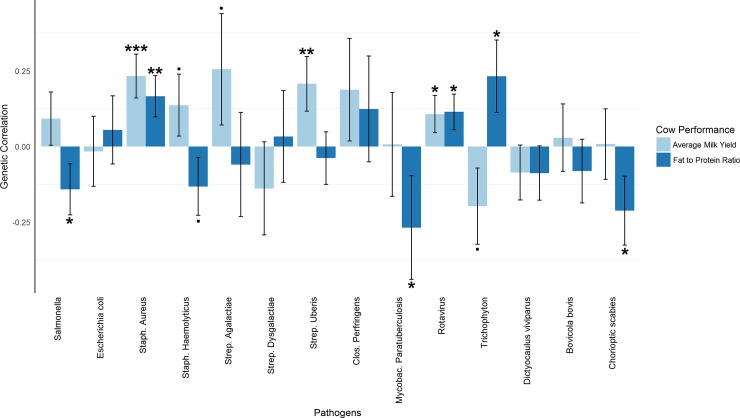
Estimated genetic correlations of average milk yield (AMY) and fat to protein ratio (FTP) with all resistance traits in cows. This plot compares the genetic correlation between AMY and all calf resistance traits with the genetic correlation between FTP and all calf resistance traits obtained from G-REML. The horizontal axis indicates pairs of phenotypes (AMY and FTP), and the vertical axis indicates genetic correlation. Error bars represent standard errors. ‘***’P ≤ 0.001; ‘**’P ≤ 0.01; ‘*’P ≤ 0.*05; ‘·’P ≤ 0*.*1*.

(Fig 4 and S5 Table). The resistance to bacterial pathogens were generally positively correlated with milk yield, the correlation being very highly significance positive (P ≤ 0.001) with Staph. Aureus, highly significantly positive (P ≤ 0.01) with Strep. Uberis and approaching significantly positive (P ≤ 0.1) with Staph. Haemolyticus and Strep. Agalactiae. Milk yield was also significantly positively correlated with resistance to Rotavirus. Resistance to Trichophyton was approaching significance for a negative correlation with milk yield. Fat to protein ratio was positively correlated with resistance Staph. Aureus (P ≤ 0.01), Rotavirus and Trichophyton (P ≤ 0.05). Fat to protein ratio was significantly negatively correlated with Salmonella, Mycobac. Paratuberculosis and Chorioptic scabies (P ≤ 0.05), and approaching significance for Staph. Haemolyticus (P ≤ 0.1).

### Biological pathway analysis

All SNPs (~50K SNPs) were annotated to the nearest gene where possible using a gene boundary extended by 20kb distance outside the transcription start site or transcription end site of the gene for all the 23 resistance traits, using MAGMA (version 1.06) and bovine gene location (UMD3.1) through ensemble-biomaRt (www.ensembl.org/biomart). This annotation pipeline resulted in a total of 16,094 genes ready for the next step of the analysis.

**Fig 5:**
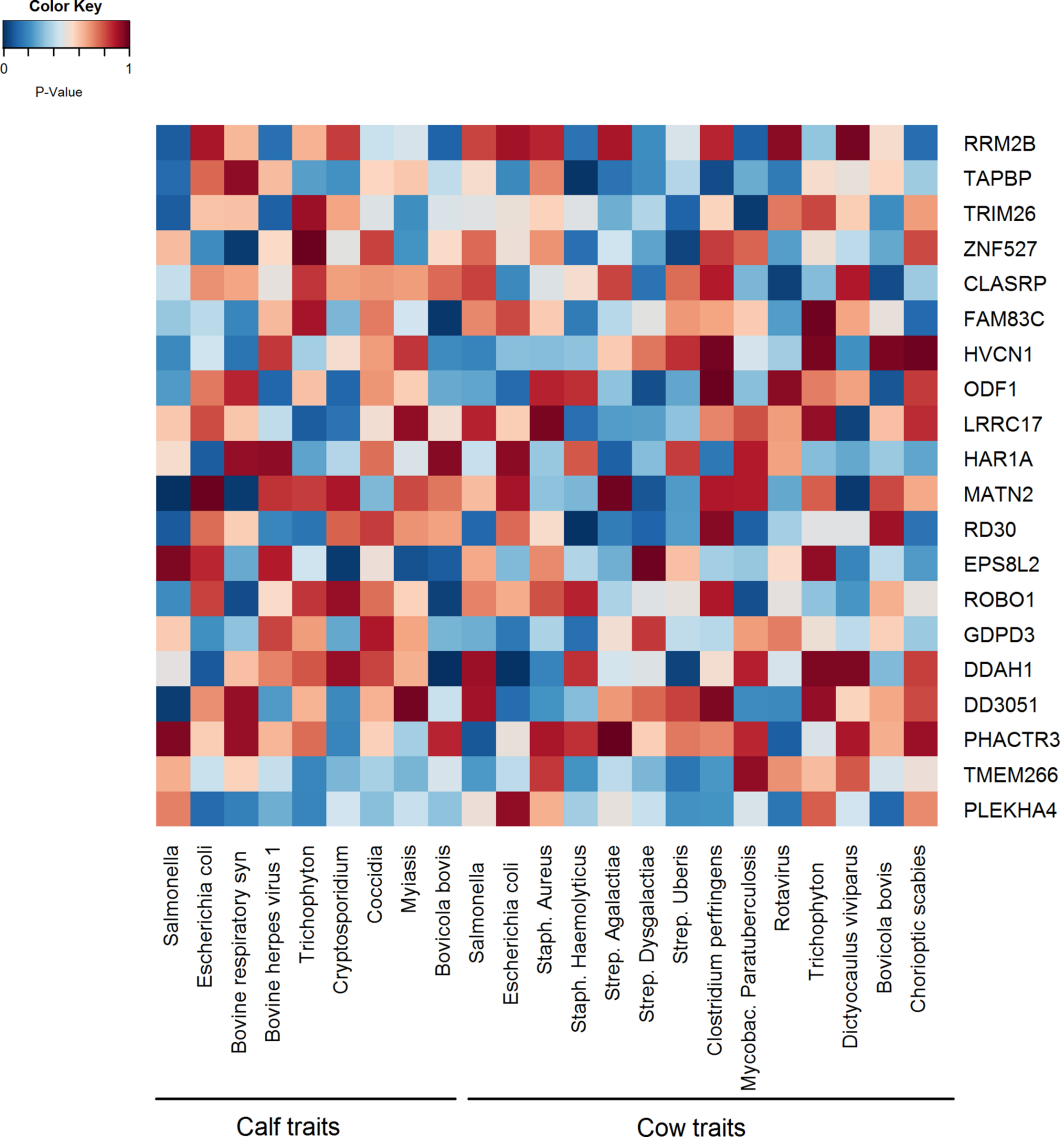
P-values for the selected annotated candidate genes in all resistance traits in calves and cows. Dark red color means very high p-value, dark blue color means very low (i.e. more significant) p-value.

**Fig 6:**
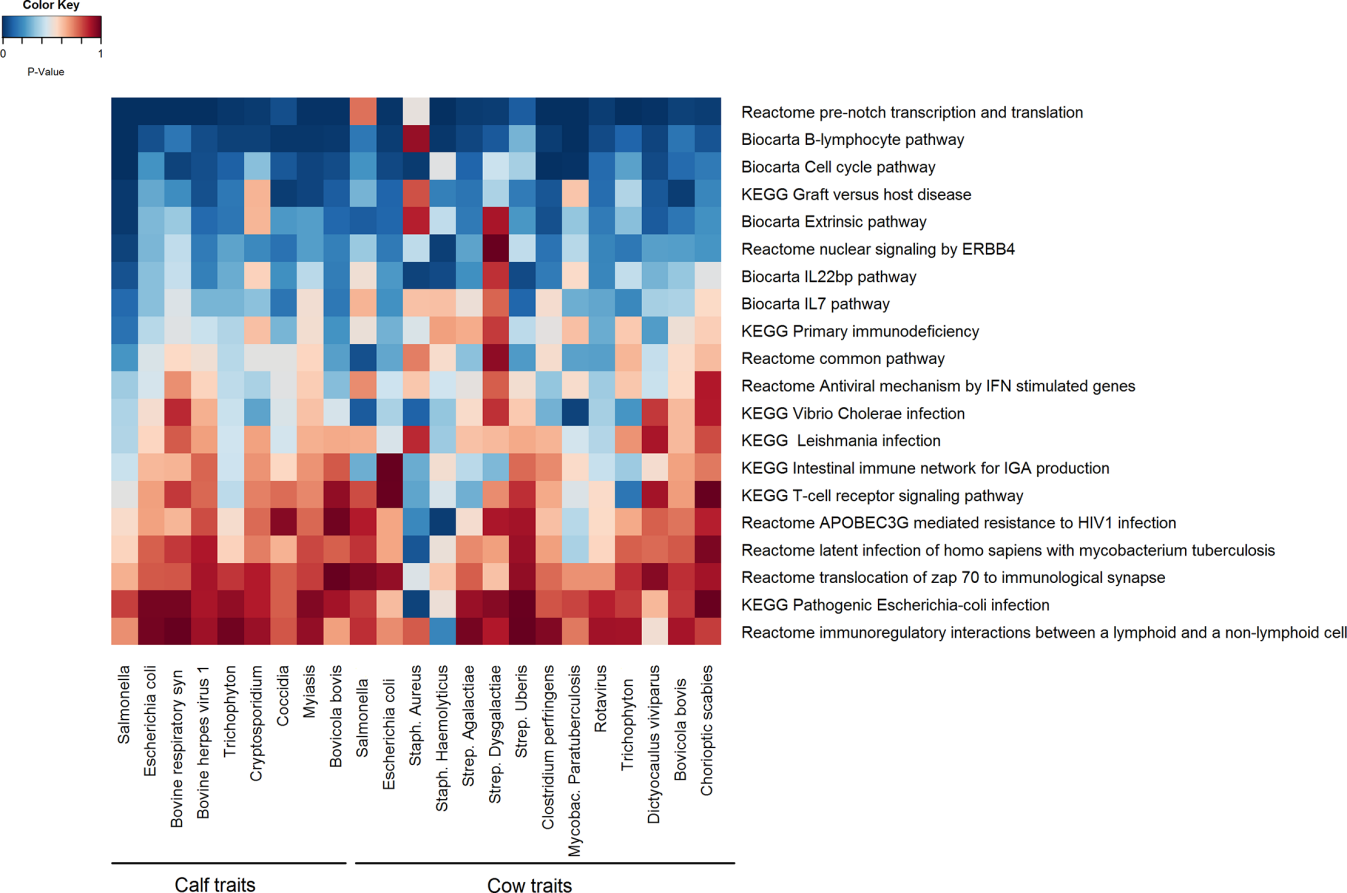
P-values for the selected 20 pathways tested across all resistance traits in calves and cows. Dark red color means very high p-value, dark blue color means very low (i.e. more significant) p-value.

**Fig 7:**
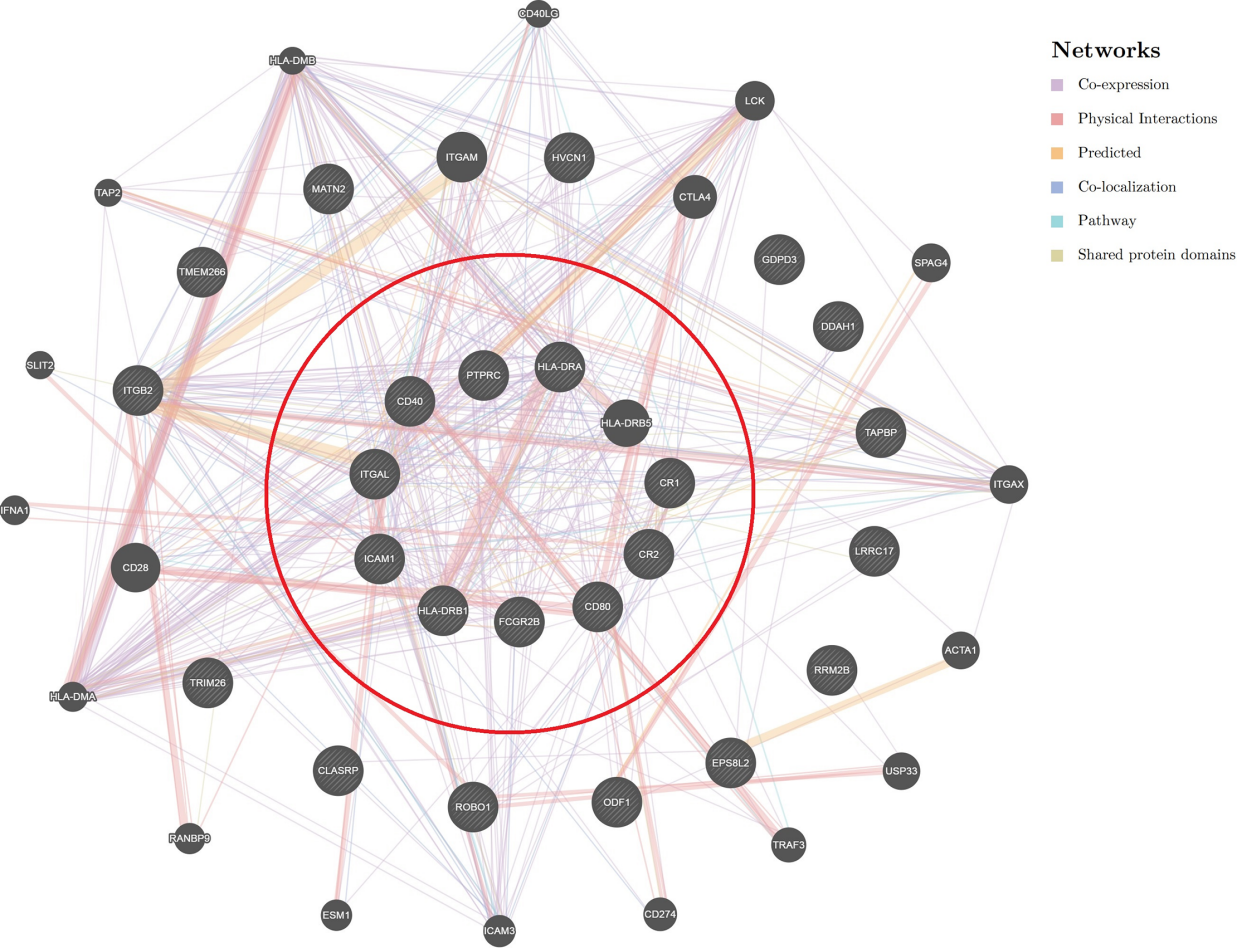
Network of gene-interactions the candidate genes (outside the red circle) and the “*Biocarta B-lymphocyte pathway*” (inside of the red circle).

After testing the association of the 16,094 genes across all the 23 resistance traits, using p-values of summary statistics of GWAS from GCTA, we selected the top (most significant) 20 genes based on average p-values. The lowest average p-value for the correlation between the genes and all resistance traits was the average p-value for the RRM2B gene (average p-value = 0.28) (Fig 5 and S6 Table). After estimating the p-values on the scale of genesets (Biological pathways), we selected the top 20 genesets based on average p-values (Fig 6 and S7 Table) out of 1083 pathways that were available from BIOCARTA, KEGG and REACTOME databases. We found that “*Reactome pre-notch transcription and translation*” and “*Biocarta B-lymphocyte pathway*” were the most associated pathways to all resistance traits according to their calculated p-values (the average p-values = 0.07 and 0.11 respectively) (Fig 6 and S7 Table). The final step was to illustrate the “*Biocarta B-lymphocyte pathway*” against the 19 genes that were associated with resistance at the gene-level. Fig 7 shows how the candidate genes (from gene analysis) were directly and indirectly connected to the “*Biocarta B-lymphocyte pathway*.”

## Discussion

We implemented G-REML using genome-wide SNP data to explore the genetic etiology of resistance to pathogens in cattle. This study thus provides the first genomic overview of the genetics of resistance across a range of pathogens together with the genetic correlations between pathogens and with production traits for Holstein cattle, the world’s predominant dairy breed. We estimated the phenotypic variance explained by all SNPs ($h_{SNPs}^{2}$) for resistance, performance and productivity traits, and calculated the genetic correlation (*r*_*g*_) between them, using genome-wide SNPs in calves and cows. The average heritability of resistance traits at a young age (calf traits) was larger than the average heritability of the same population at a later age (cow traits), reflecting the increase of the environmental effect potentially including increased pathogen exposure after first calving in cattle. The SNP heritabilities estimated using GREML in this study were of a similar scale to those for which estimates for individual pathogens have been previously published using pedigree and genome-wide data [6, 7].

### Pleiotropy between resistance to pathogens and calf performance and cow productivity

There have only been very limited previous estimates of genetic correlations among resistance to different pathogens with which our estimates can be compared (e.g. [37]). We thus report a number of new findings that would be difficult to obtain from pedigree studies, including some very high estimates of positive pleiotropy between the resistance to bacterial, fungal and parasite pathogens. Negative pleiotropy was found between the resistance to most of the bacterial, viral and fungal pathogens with both performance traits that we analysed in calves (birthweight and average daily gain). Results supports the hypothesis that some resistance genes may negatively impact performance traits in young calves, perhaps reflecting a balance between energy expenditure on disease resistance and growth. However, clear positive pleiotropy found between resistance to bacterial, viral and fungal pathogens with average milk yield in cows supports the hypothesis that cows that are genetically less vulnerable to infections can produce more milk. This provides economic in addition to welfare justification for increasing focus of breeding objectives on these disease resistance traits.

### Shared biological pathways

To date, few studies have implemented biological pathway analysis in animal health [38, 39]. This is the first study post-GWAS for resistance to most of all infection pathogens in dairy cattle. We found a group of 20 genes shared effects across all resistance traits and showed that “*Reactome pre-notch transcription and translation*” and “*Biocarta B-lymphocyte pathway*” are the most consistently associated pathways with resistance to different pathogens. The genetic correlations that we observed in this study show a clear pleiotropy (by the means of similar resistance mechanism against the 23 pathogens). The combination of accurate recording of multiple diseases and associated pathogens combined with associated genomic data is unusual and possibly so far unique in a mammalian species including humans. However, given the abundance of genomic data in humans combined with GWAS for a number of individual infectious diseases analyses such as performed here should become increasingly possible for human populations.

The scale of this dataset facilitated by the thorough electronic data collection within the dairy cattle test-herd system in north-eastern Germany has enabled us to obtain unique insight into the genetics of resistance across a range of pathogens. The corollary of these currently unique data is that the overall multivariate pattern will need to await further data collection from this or other similar programs before it is possible to replicate our results as a whole. Nonetheless, all significant correlations have a low standard error (S3, S4 and S5 Tables), and our estimates of heritabilities and pairwise genetic correlations are generally consistent with others in the literature where these are available (e.g. [6, 7, 37, 40]).

### The biological impact of the genetic pleiotropy on breeding and selection strategies

The estimated heritabilities and the pattern of genetic correlations between pathogens and with production traits provide valuable information allowing the further optimisation of cattle breeding programmes. For example, several low and non-significant genetic correlations were found among calf resistance traits as well as among cow resistance traits, questioning the traditional hypothesis of selection for mastitis resistance based on somatic cell count as a consequence of multiple pathogen infection in cattle [41] or in sheep [42]. Additionally, the highly significant negative correlation between resistance to the Salmonella pathogen in calves and cows implies that resistance traits in calves are not good indicators or early predictors for the resistance traits and genetic improvement of cow health after calving in first parity, supporting a previous pedigree and GWA study [40]. The estimated zero genetic correlation between resistance to the Trichophyton pathogen in calves and cows indicates that, for this pathogen, calf and cow resistance, are genetically distinct. Thus, overall it is clear that to design an efficient breeding program, we need to take into account these results and utilise a programme combining selection in both cows and calves and consider how to most effectively collect and incorporate information on resistance and susceptibility to multiple pathogens. Fortunately, the increasing availability of genomic data in cattle combined with collection of data such as analysed here ultimately will facilitate genomic selection programmes that meet these objectives.

**In summary,** despite some limitations of available health traits and genotyped data, the use of G-REML method to estimate the genetic correlation among health traits and between health trait and performance and productivity traits promises to be a very valuable tool in the genetic improvement of animal health. Biological pathway analysis appears to be a very useful tool also, but at present we have had to use information for other species as have no biological pathways specifically tested and verified for cattle and the development of such databases would provide an invaluable resource for future research.

## Materials and methods

The resistance to infectious disease traits in Holstein cattle was measured through the framework of the dairy cattle test-herd system of northeast Germany, including the federal states of Mecklenburg-Westpommerania and Berlin-Brandenburg. Dairy cattle farmers and veterinarians used electronic recording systems, which were based on the diagnosis key as developed by Feucker and Staufenbiel [43]. This diagnosis key was also considered when developing the International Committee for Animal Recording (ICAR) [44].

### Phenotypes

In calves and cows, four main domains of pathogens were used to classify 23 resistance traits: (a) bacterial pathogens, (b) viral pathogens, (c) fungal pathogens, and (d) parasitic pathogens. We distinguished between calf resistance traits and cow resistance traits [40]. For the traits recorded in calves, we defined a time window from birth to the age of 150 days. For the traits recorded in cows, the window was from 20 days before first calving up to 365 days after first calving (a 385-day period). At least one entry for the respective pathogen implied a score = 0 for infected (non-resistant); otherwise, score = 1 for non-infected (resistant). The infected animal with a given pathogen was the animal (calf/cow) that was recorded as infected with this pathogen during its calf/cow age. A non-infected (resistant) calf to a given pathogen was defined as a calf that was found to be healthy on a farm infected with the given pathogen and that was born after the first record of this infection on that farm; a resistant cow to a given pathogen is defined as a cow that was found to be healthy in a farm where the first record of infection with the given pathogen was at least 20 days before its first calving date. Note that in common with other studies of natural infection in livestock and other species, we use resistance to define animals that did not become infected in a herd that was undergoing a specific disease challenge. For a given pathogen, all herds that showed no occurrence of infection were excluded because we do not know whether these herds were challenged with the particular pathogen.

All veterinary diagnosis and infection pathogen recording was done according to the ICAR (S8 Table, also available online through: www.icar.org). The nine pathogen resistance traits that were recorded in calves were Salmonella, Escherichia coli, Bovine respiratory syn., Bovine herpesvirus 1, Trichophyton, Cryptosporidium, Coccidia, Myiasis and Bovicola bovis. The 14 pathogen resistance traits that were recorded in cows were Salmonella, Escherichia coli, *Staphylococcus aureus* (Staph. Aureus), *Staphylococcus haemolyticus* (Staph. Haemolyticus), *Streptococcus agalactiae* (Strep. Agalactiae), *Streptococcus dysgalactiae* (Strep. Dysgalactiae), *Streptococcus uberis* (Strep. Uberis), *Clostridium perfringens* (Clost. Perfringens), *Mycobacterium avium paratuberculosis* (Mycobac. Paratuberculosis), Rotavirus, Trichophyton, Dictyocaulus viviparous, Bovicola bovis and Chorioptic scabies. For testing the genetic correlation between the resistance and performance in calves we considered two performance traits in calves: birthweight (in kg) and average daily gain (in g/day) during the first 360 days of calf life. For testing the genetic correlation between the resistance and productivity in cows, we considered two productivity traits in cows: average milk yield (in kg) during the first lactation and fat to protein ratio (in %) during the first lactation.

### Genotypes

Genotyping was performed using the Illumina Bovine 50K SNP-BeadChip V2 (Illumina Inc., San Diego, CA), and with the Illumina Bovine Eurogenomics 10K low-density chip. The low-density genotypes (10K) were imputed by Vereinigte Information system Tierhalung (Verden, Germany) to the 50K panel applying the algorithm by Segelke et al. [45]. In post-imputation SNP quality checks; animals with almost identical SNP genotypes (>95% congruency across all SNPs) were eliminated from the ongoing analyses; SNPs with minor allele frequency <0.01 and SNPs showing a significant (P < 10^−5^) deviation from Hardy-Weinberg equilibrium were discarded. All SNPs had a genotype call rate greater than 95%.

Genotyping was only undertaken for infected and resistant animals from populations where we could be sure that all animals genotyped have been challenged with the relevant pathogen (i.e. that they come from an infected population). Hence, the incidences from these genotyped samples do not reflect the actual incidences in the German Holstein population, but are likely to be higher as we have excluded data from herds where there is no evidence for a disease challenge for a particular pathogen. Further epidemic research, it is required to study the true incidence at the population scale. For a full statistical descriptive of the 23 resistance traits and the four quantitative traits in calves and cows in the genotyped sample, see Table 1.

## Statistical models

### Correction for population stratification in genome-wide data

The most common method for dealing with population stratification is principal component analysis (PCA) [46, 47]. Fitting the leading principal components in the model can correct for stratification for analyses such as estimating the proportion of variance explained by genome-wide SNPs and for genome-wide association studies (GWAS). Here, we applied principal components analysis to the genome-wide SNP data to infer continuous axes of genetic variation. Hence, the new axes will reduce the data dimensions (eigenvectors), describing as much variability as possible (eigenvalues): *V*^−1^(*c*^*t*^*c*)*V* = *D*, where ***V*** was the matrix of eigenvectors which diagonalizes the covariance matrix ***c***^*t*^***c*** (covariance matrix of genotyped data), ***D*** was the diagonal matrix of eigenvalues of ***c***^*t*^***c***. Then, we adjusted the phenotypes by including the first five eigenvectors as covariates in the model when estimating the proportion of variance explained by all the SNPs, or in G-REML and GWAS (see model-1).

A univariate mixed linear model was used to estimate the phenotypic variance explained by all autosomal SNPs ($h_{SNPs}^{2}$) by applying the genomic-restricted maximum likelihood analysis (G-REML), and using the GCTA software [48]. In matrix notation, the model was defined as:

$$y=Xb+g_{G}+\varepsilon$$ (1)

where **y** refered to the vector of the quantitative trait (for performance and productivity traits) or of unobserved liabilities (for resistance traits); **b** was the vector of the fixed effects (herd, birth year and birth month for calf traits; herd, calving year, calving season, age at first calving for cow traits and the first 5 PCs), **X** was an incidence matrix for the fixed effects; ***g_G_*** was the vector of aggregated effects of all autosomal SNPs with $var(g_{G})=A_{G}\sigma_{G}^{2}$ and *A*_*G*_ was the genomic relationship matrix (GRM). The heritability explained by all autosomal SNPs ($h_{SNPs}^{2}$) was defined as $h_{SNPs}^{2}=\sigma_{SNPs}^{2}/\sigma_{p}^{2}$ where $\sigma_{p}^{2}$ was the phenotypic variance.

A bivariate model was used to estimate the genetic correlation among resistance traits and between resistance and performance or productivity traits [48].

$$\left[\begin{array}{c} y_{1}=X_{1}b_{1}+g_{G1}+e_{1}\;(trait\;1)\\ y_{2}=X_{2}b_{2}+g_{G2}+e_{2}\;(trait\;2) \end{array}\right]$$ (2)

The two equations are the same as in model 1, while the (co)variance matrix was:

$$var\left[\begin{array}{c} y_{1}\\ y_{2} \end{array}\right]=\left[\begin{array}{cc} \sigma_{G_{1}}^{2}A+\sigma_{e_{1}}^{2}I & \sigma_{G_{1}G_{2}}A+\sigma_{e_{1}e_{2}}I\\ \sigma_{G_{1}G_{2}}A+\sigma_{e_{1}e_{2}}I & \sigma_{G_{2}}^{2}A+\sigma_{e_{2}}^{2}I \end{array}\right]$$ (3)

where **A** is GRM, **I** is the identity matrix, $\sigma_{G}^{2}$ is the genetic variance, $\sigma_{e}^{2}$ is the residual variance and $\sigma_{G_{1}G_{2}}$ is the genetic covariance between the two traits. We assumed that all environmental correlations between pathogens were zero, as less than 10% of animals shared diagnoses for any pair of pathogens.

### Significance thresholds

To determine the significance of the estimated pleiotropy among calf and cow resistance traits, two methods are commonly used to determine the significance threshold for genome-wide analysis: the false discovery rate and Bonferroni correction. The False discovery rate (FDR) correction was introduced by Benjamini and Hochberg [49]. The FDR method first ranks all p-values from the smallest to the largest, and then adjusts each p-value accordingly:

$$FDR\;corrected-p=\frac{Number\;of\;tests}{p-vlaue\;ranking}*p-value$$

For the pleiotropy among resistance traits, we used the FDR of 1%. Bonferroni correction bases on the number of independent tests performed in each scenario (36 tests in calf traits and 105 tests in cow traits). The Bonferroni threshold was used at *α* = 1%, and calculated as follows:

$$Bonferroni\;threshold=-log10(\frac{\alpha }{Number\;of\;tests})$$

### Biological pathway analysis

Biological pathway analysis is an approach where the association between a select set of genes (biological pathways) and a trait of interest (the resistance to different pathogens) was tested. This analysis can be used to test the cumulative genetic effects across multiple genes within a pathway.

### Multi-marker Analysis of GenoMic Annotation (MAGMA)

MAGMA used in our pathway analysis according to Leeuw et al., [50] with the following three steps: First, an annotation step to map SNPs onto genes using the bovine gene location (UMD3.1), from ensemble-biomaRt (www.ensembl.org/biomart). Second: a gene-based analysis step to compute gene p-values, using MAGMA and the output of GWAS from GCTA (pre-estimation of summary statistics of GWAS for each trait, performed using the GCTA software [48]. Third: a gene-set (Biological pathways) analysis step, to compute biological pathway p-values, using MAGMA (with the “*competitive gene-set analysis”* function) with publicly available BIOCARTA, KEGG and REACTOME database. All analyses in MAGMA are structured as a linear regression model on gene-level data.

$$Z=\beta_{0}+\beta_{1}G_{1}+e$$ (Model – 4)

Where ***Z*** was the phenotype vector, Gene-sets ***G***_*1*_ were binary indicator variables, coded with “1” for genes in the gene-set, and with “0” otherwise. e was the residual vector. The intercept *β*_*0*_ represents the mean, and *β*_*1*_ the association specific to the gene-set 1. One last step was to illustrate the most significant gene rich network related to most of the resistance traits in calf and cows. This was done using the web-based software GeneMANIA [50], and then to illustrate the most significant related genes to all resistance traits (both in the same Fig) to see how these genes are related to this pathway. The post-GWAS functional analyses were performed using the MAGMA software [50] and the GWAS output from GCTA software [48].

## Supporting information

S1 Table (3 sheets). **correlations:** Genetic correlations among the 9 calf traits analyzed by G-REML**p-value:** p-values for genetic correlations among the 9 calf traits analyzed by G-REML**se:** Standard error for genetic correlations among the 9 calf traits analyzed by G-REML.
(XLSX)

S2 Table (3 sheets). **correlations:** Genetic correlations among the 14 cow traits analyzed by G-REML**p-value:** p-values for Genetic correlations among the 14 cow traits analyzed by G-REML**se:** Standard error for Genetic correlations among the 14 cow traits analyzed by G-REML.
(XLSX)

S3 Table Genetic correlations between resistance to the same pathogen in calves and cows analyzed by G-REML. (XLSX)

S4 Table Genetic correlations between all calf resistance traits and either birth weight or average daily gain in calves. (XLSX)

S5 Table Genetic correlations between all cow resistance traits and either milk yield or fat to protein ratio. (XLSX)

S6 Table P-values for the candidate 20 genes and their average across all resistance traits in calves and cows. (XLSX)

S7 Table P-values for the most important 20 pathways and their average across all resistance traits in calves and cows. (XLSX)

S8 Table Summarizes the code of all pathogens. (XLSX)
